# Coronavirus Disease (COVID-19) in Italy: Analysis of Risk Factors and Proposed Remedial Measures

**DOI:** 10.3389/fmed.2020.00140

**Published:** 2020-04-09

**Authors:** Giuseppe Di Lorenzo, Rossella Di Trolio

**Affiliations:** ^1^Medical Oncology, Andrea Tortora Hospital, ASL Salerno, Pagani, Italy; ^2^Department of Medicine and Health Sciences Vincenzo Tiberio, University of Molise, Campobasso, Italy; ^3^Unit of Melanoma, Cancer Immunotherapy and Development Therapeutics, Istituto Nazionale Tumori IRCCS Fondazione G. Pascale, Naples, Italy

**Keywords:** coronavirus, causes and management, lung disease, potential drugs, prevention

## Introduction

Until March 28, 2020, there were ~90,000 confirmed cases of coronavirus disease (COVID-19) in Italy, with 26,000 in-patients, 3,800 patients in intensive care units (ICUs), 40,000 positive in home isolation, and 10,000 deaths, according to the Italian Civil Protection bulletin^1^. Italy currently has the highest COVID-19 mortality rate worldwide, even compared to the People's Republic of China where the number of COVID-19 deaths totaled over 3,000 cases, including potential re-infections. Globally, there are ~570,000 cases and 26,000 deaths due to COVID-19. According to the World Health Organization (WHO) as of March 28, 2020, the number of COVID-19 positive cases in Spain is increasing, with 64,000 infected cases and 5,000 deaths.

In the United States, in the span of a few days, there were 85,000 cases and 1,200 deaths due to COVID-19; Germany has 48,000 confirmed cases, and France has 32,000 cases with 600 deaths^2^.

After a short respite with 9,000 COVID-19 cases and only 140 deaths, the infection has resurged and the number of confirmed cases are continuously increasing in South Korea^3^. An analysis of the data in the daily updates communicated by the Civil Protection, showed that most of the COVID-19 cases and deaths are limited to Northern Italy—especially Lombardy, Emilia Romagna, Veneto, and Piedmont—with the numbers fortunately decreasing toward central and southern Italy, and very few cases documented in Basilicata^1^. The most severely affected regions are also the regions where healthcare services have always been considered excellent; the hospitals of Lombardy and Veneto are the Italian centers of excellence with regard to standard protocols and management for many diseases, especially neoplastic conditions, and there was a high rate of passive migration of patients from the South to northern hospitals.

The Lombardy region has a higher number of intensive care and resuscitation beds compared to southern Italy; unfortunately, these places are fast running out of hospital beds and facing challenges in the provision of primary care for conditions other than COVID-19, necessitating the transfer of numerous patients to other regions^4^. The situation would probably have been considerably worse if the regions of Southern Italy had the highest number of COVID-19 cases.

These numbers confirm the fact that we are facing a pandemic, which was declared by the WHO a few days ago.

### Why Are There So Many Cases and So Many Deaths in Italy?

The lethality rate is determined as follows: the number of deaths due to COVID-19 divided by the total number of confirmed coronavirus cases. In Italy, the lethality rate is 9%, which peaks in Lombardy (>10%), whereas the lethality rate in Wuhan was 5.8% and remained <1% in the rest of the People's Republic of China^4^. An initial rationale for the higher lethality rate could be the high average age of the Italian population when compared to, for example, the People's Republic of China and the Republic of Korea; in the latter, the majority of confirmed COVID-19 cases are young women (62%), with 30% of positive cases in the age range of 20–30 years. The average age of those dying in Italy is 79 years, and more than 70% were men^1^.

Another explanation for the higher lethality is the presence of other pathologies and the comorbidities of the elderly population. Based on research by the WHO, a Report of the WHO China Joint Mission on Coronavirus Disease 2019, published in February 2020, reported that patients without other comorbidities have mortality rates of 1.4%, compared to COVID-19 patients with other diseases that compromise their health condition and result in higher mortality rates, which were 13, 9, and 7.6% for those with cardiovascular disease, diabetes, and cancer, respectively^5^. In Italy, data from the Istituto Superiore di Sanità (ISS) indicates that 1% of the patients who died had no other disease, 26% had 1 disease, 26% had 2 disease, and 47% had 3 or more conditions. The most common chronic preexisting disease in the patients who died was arterial hypertension (76%), followed by ischemic heart disease (37%), atrial fibrillation (26%), and active cancer within the previous 5 years (19%)^4^.

Another cause for the higher lethality rate may be that Italy had a higher number of infected individuals who were asymptomatic and infected others. As recently reported by Li et al., the transmission rate from unreported infections was 55% of the rate of reported infections, and un- reported infections resulted in 79% of reported cases (1). Therefore, for each positive COVID-19 case, there are ~8–10 undetected cases; thus, the actual number of COVID-19 cases could be up to 10 times higher, and recalculation of the mortality rates on this basis would cause the actual national mortality rate of COVID-19 to decrease approximately to the mortality rates of COVID-19 in the People's Republic of China.

In Lombardy, there is a considerable amount of business travel and many people work in hospitals, which could have amplified the infection spread. In fact, doctors and nurses constitute the most infected occupational categories. Moreover, at the beginning of the epidemic in Lombardy, especially in Bergamo, many patients had visited general practitioners who had no experience with the new virus. Several of these doctors have been infected and have, unfortunately, died.

It also cannot be ignored that the elderly in Italy have frequent contact with their children and often take care of grandchildren. The percentage of people between the age of 30 and 49 years who live with their parents is up to 20%, which is much higher than in other countries. Adult children and grandchildren, who are often asymptomatic, would have infected their elderly parents.

## Discussion

### What Remedial Steps Can Be Undertaken?

1. **Prevention**. Certainly, as has been reiterated by the Italian government several times in the previous 2 weeks, it is necessary to limit the infection spread by not going out unless for work. On March 21, the Prime Minister, Giuseppe Conte, announced the closure of all non-essential production activities. However, activities essential to guarantee essential goods and services continue to remain operational. The Minister of Health has passed a new ordinance that increases the restrictions imposed on citizens wherein outdoor activities and visits to parks and gardens are prohibited^7^. Furthermore, restaurants and bars had already been closed until the end of March 2020^6^. Some regions of southern Italy have passed ordinances that prohibit, with immediate effect and until April 14, 2020, any movement of persons entering and leaving these regions. For example, one can only enter or leave the Calabria and Campania Regions for journeys deriving from verified essential requirements related to the provision of essential services or for serious health reasons. In light of the potential exposure to infection, an immediate measure of a 14-day quarantine will apply for those who violate these restrictions^8^.
2. **Increase ICU beds and create new hospitals**. For this, the President of the Campania Region, Vincenzo De Luca, worried about the deterioration in southern Italy, has announced the forthcoming construction of two new modular hospitals in Napoli and Caserta. They will be a sort of “field” hospital, consisting of containers and blocks which will form the body of the hospitals, with 48 added beds; other intensive care places were obtained by reconverting hospitals, in some cases ones that were previously closed. Three thousand additional beds will be recovered from private clinics^9^.
3. **Increased number of doctors and nurses for the northern regions**. On March 21, a vacancy call for 350 doctors was published. These new doctors, in coordination with the Civil Protection, will likely be included in task groups. Within 24 h, there were more than7,900 enquiries and Italian doctors were joined by doctors from other countries, such as the People's Republic of China and Cuba^1^.
4. **Increased supply of masks and mechanical ventilators**. The Ministries of Internal Affairs and Foreign Affairs have announced that ~3 million masks and 300 mechanical ventilators have been procured from many countries^4^.
5. **Increased testing for asymptomatic people, particularly those exposed on the frontlines such as doctors and nurses**. The mortality rate in Italy is higher because asymptomatic cases are not being tested and isolated. At the beginning of the epidemic, there was misinformation that asymptomatic cases did not transmit the virus. This statement is certainly incorrect, and the recognition of asymptomatic/mildly symptomatic cases could decrease the number of infections (1). Numerous regions of Central and Southern Italy have communicated a decision to screen doctors and nurses even if they are asymptomatic^6^^,^^9^.
  According to the Campania Region official press release n° 96 on 30th March, 2020, referring to decree n°45 on 6th March, 2020, serological tests will also be done on patients in pre-triage. Serological tests are quick qualitative tests which research antibody IgM or IgG anti corona virus' antigens^9^.
6. **Completely ban smoking**. Given that the most serious outcome of COVID-19 is pneumonia, the number of deaths could reflect the presence of fine dust in the air, especially in Lombardy, and the state of the average Italian lungs, which are damaged by cigarette smoke. The fact that more men have died may be attributed to a smoking habit, and those who smoke are more likely to become seriously ill with COVID-19.
7. **Protect and monitor patients with comorbidities**: These patients are at a greater risk of infection with severe acute respiratory syndrome coronavirus 2 (SARS-CoV-2), and cancer patients should consider postponing adjuvant treatments, elective surgical interventions, or follow-up visits where possible^9^. To date, there is no vaccine for COVID-19, and it might take several months for any new vaccine to be developed.
8. **Therapies**. Various drugs have been used, including antivirals (e.g., favipiravir, arbidol, remdesivir) and antimalarials (e.g., chloroquine) (2), or tocilizumab in patients with high levels of interleukin 6 (IL6) and extensive bilateral pulmonary lesions or severe symptoms. The Italian Drug Agency (AIFA) has announced the authorization of the TOCIVID-19 study, which will assess the efficacy and safety of tocilizumab in the treatment of pneumonia in COVID-19. The above-mentioned study will evaluate the impact of tocilizumab (approved for rheumatoid arthritis), which has recently been reported to have conferred possible benefits on patients treated by Dr. Paolo Ascierto. In the TOCIVID-19 trial, 330 hospitalized COVID-19 patients with pneumonia with early signs of respiratory failure or who were intubated and placed on ventilatory treatment within the previous 24 h will be treated with tocilizumab^10^.

AIFA has also authorized 2 additional trials: the first trial is the combination of emapalumab, monoclonal antibody anti-gamma interferon with Anakinra, an IL-1 antagonist; in the second trial Sarilumab, an IL6 antagonist, will be used^10^.

*In vitro* studies have shown that nitric oxide (NO) inhibits the replication of SARS-CoV-2. Recently, the U.S. Food and Drug Administration (FDA) has granted Bellerophon Therapeutics an expanded access to enable the use of inhalational NO delivery systems in COVID-19 patients^11^.

Furthermore, tests that are faster than the current nasopharyngeal swab or serum tests would be desirable, especially in the asymptomatic population. Recently, the US FDA has approved the first coronavirus diagnostic test that can be conducted entirely at the point of care. The test will deliver results within 45 min, which is much faster than current tests that require a sample to be sent to a centralized laboratory, which can take days for results to be reported^12^.

Unfortunately, in recent years, investments in healthcare and research have been limited in Italy. Public healthcare expenditure in 2018 represented 6.5% of the gross domestic product (PIL), which is much lower than that of other countries such as France and Germany; many Italians have turned to the private sector (+16%) from the public sector in the last 2 years. However, this is not the time for recriminations and airing of political differences.

The battle ahead is long and will not end in a few weeks. The lifestyle and habits of Italians have changed. To date, ~3,800 ICU beds are occupied, and, as reported by Remuzzi and Remuzzi (3), up to 4000 hospital beds will be needed by April 2020; therefore, all regions must prepare or secure additional beds. We must absolutely avoid a collapse of the healthcare system and having to choose who to cure and who to let die.

At the moment, the best weapon against COVID-19 is strict adherence to the rules. Among other things, one should avoid social assembly, maintain interpersonal distances of at least 1 m, possibly 2 m, and thoroughly wash their hands often.

## Author Contributions

All authors listed have made a substantial, direct and intellectual contribution to the work, and approved it for publication.

### Conflict of Interest

The authors declare that the research was conducted in the absence of any commercial or financial relationships that could be construed as a potential conflict of interest.

## Footnotes

^1^http://www.protezionecivile.gov.it/ (accessed March 28, 2020).

^2^https://www.who.int/health-topics/coronavirus#tab=tab_1 (accessed March 28, 2020).

^3^https://www.scmp.com/topics/coronavirus-south-korea (accessed March 23, 2020).

^4^http://www.salute.gov.it/nuovocoronavirus (accessed March 28, 2020).

^5^https://www.who.int/docs/default-source/coronaviruse/who-china-joint-mission-on-covid-19-final-report.pdf (accessed March 25, 2020).

^6^http://www.governo.it/ (accessed March 28, 2020).

^7^http://www.governo.it/it/articolo/ordinanza-del-ministro-della-salute-20-marzo-2020/14355 (accessed March 25, 2020).

^8^https://www.regione.calabria.it/website/portaltemplates/view/view.cfm?17173 (accessed March 30, 2020).

^9^http://www.regione.campania.it/assets/documents/ord-n-16-13-03-2020.pdf (accessed March 30, 2020).

^10^www.aifa.gov.it/emergenza-covid-19 (accessed March 30, 2020).

^11^https://bellerophon.gcs-web.com/news-releases/news-release-details/fda-grants-bellerophon-emergency-expanded-access-inopulser (accessed March 25, 2020).

^12^https://www.fda.gov/emergency-preparedness-and-response/mcm-issues/coronavirus-disease-2019-covid-19 (accessed March 25, 2020).

## References

[1] Li R Pei S Chen B Song Y Zhang T Yang W. Substantial undocumentedinfectionfacilitates the rapiddissemination of novelcoronavirus (SARS-CoV2). Science. (2020) eabb3221. 10.1126/science.abb3221 32179701 PMC7164387

[2] Dong L Hu S Gao J. Discovering drugs to treat coronavirus disease 2019 (COVID-19). Drug Discov Ther. (2020) 14:58–60. 10.5582/ddt.2020.01012 32147628

[3] Remuzzi A Remuzzi G Covid 19 and Italy. What next? Lancet. (2020) 20:30627–9. 10.1016/S0140-6736(20)30627-9 PMC7102589 32178769

